# Understanding the Importance of Capsules in Dry Powder Inhalers

**DOI:** 10.3390/pharmaceutics13111936

**Published:** 2021-11-16

**Authors:** Francesca Buttini, Eride Quarta, Chiara Allegrini, Federico Lavorini

**Affiliations:** 1 Food and Drug Department, University of Parma, Parco Area delle Scienze 27/A, 43124 Parma, Italy; eride.quarta@studenti.unipr.it; 2 Department of Experimental and Clinical Medicine, University of Florence, Largo Brambilla 3, 50134 Florence, Italy; chiara.allegrini.med@gmail.com (C.A.); federico.lavorini@unifi.it (F.L.)

**Keywords:** capsule, dry powder inhaler, inhalation, pulmonary drug delivery

## Abstract

Pulmonary drug delivery is currently the focus of research and development because of its potential to produce maximum therapeutic benefit to patients by directing the drug straight to the lung disease site. Among all the available delivery options, one popular, proven and convenient inhaler device is the capsule-based dry powder inhaler (cDPI) for the treatment of an increasingly diverse range of diseases. cDPIs use a hard capsule that contains a powder formulation which consists of a mixture of a micronized drug and a carrier usually the lactose, known for its good lung tolerance. The capsule is either inserted into the device during manufacturer or by the patient prior to use. After perforating, opening or cut the capsule in the device, patients take a deep and rapid breath to inhale the powder, using air as the vector of drug displacement. The system is simple, relatively cheap and characterized by a lower carbon footprint than that of pressurized metered dose inhalers. This article reviews cDPI technology, focusing particularly on the importance of capsule characteristics and their function as a drug reservoir in cDPIs.

## 1. Introduction

Pressurized metered-dose inhalers (pMDIs), dry powder inhalers (DPIs) and nebulizers are the main categories of inhaled drug delivery systems, each class with its unique strengths and weaknesses [1]. This classification is based on the physical state of the formulation as well as on the type of device used to meter, deliver and aerosolise the dose of product to the lungs. pMDIs and DPIs, containing a suspended or dissolved drug in a propellant or a drug as in dry powder form, are the most widely used drug delivery systems for lung disease treatment. The delivery of pharmacological agents by inhalation is a critical issue in obstructive airway diseases such as asthma and Chronic Obstructive Pulmonary Disease (COPD).

The development of the first commercially available inhaler, in the form of a pMDI, dates back to 1956. Although this was an innovation for the therapy of pulmonary diseases, concerns arose around the 1970s when the contribution of chlorofluorocarbon (CFC) propellants to the depletion of the ozone layer led to their substitution by more environmentally friendly hydrofluoroalkane (HFA) gases, still used nowadays [2]. These inhalers generate a drug aerosol upon actuation and the drug is suspended or solubilized in the propellant. In the case of suspension pMDIs, the high variability and inconsistency of the emitted dose when the inhalers are not shaken properly suggest the importance of following the leaflet instructions and of training the patient on this topic [3]. Moreover, the coordination between the device actuation and inhalation is a key element for the efficacy of particle deposition in the lung and for the overall disease treatment.

**Figure 1:**
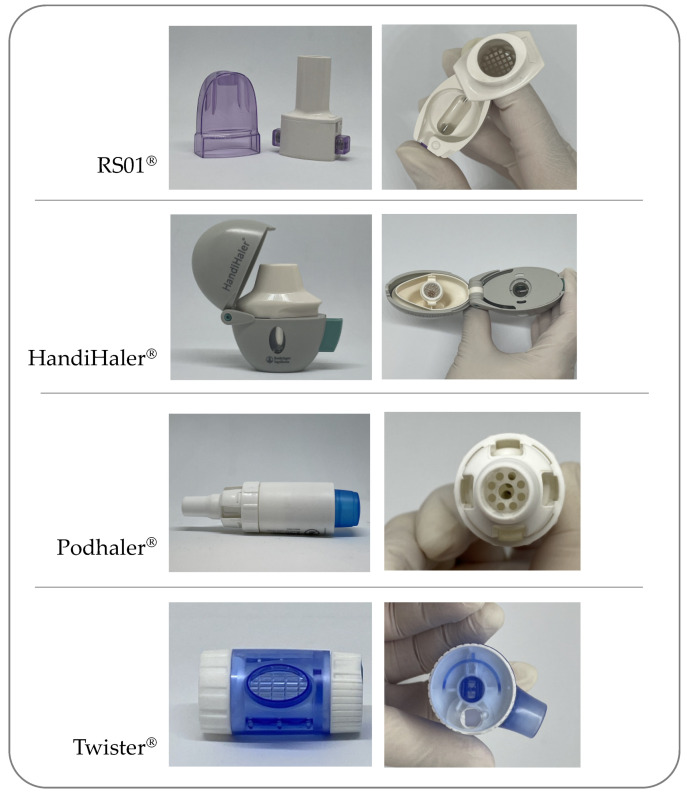
Different types of capsule-based dry powder inhalers with corresponding capsule perforation or opening mechanism; from the top: RS01, HandiHaler, Podhaler and Twister.

With the aim of overcoming pMDI limitations, DPIs were developed and for the first time placed on the market in the late 1960s, when Fisons (Ipswich, UK) developed the Spinhaler^®^ device, which used two pins to create opposing holes in the sidewall of the body of a gelatin capsule loading the powder dose [4]. Subsequent DPIs have used either pairs of pins, to make single holes in the sidewalls (HandiHaler^®^, Boehringer Ingelheim, Germany) or in the domed ends (RS01^®^ Plastiape, Lecco, Italy; Breezhaler^®^ Novartis, Basel, Switzerland; Turbospin^®^ PH&T, Milan, Italy), or two sets of four pins (RS00 Plastiape, Lecco, Italy; Aerolizer^®^ Novartis, Basel, Switzerland), to make multiple holes in both domed body and cap. In each of these devices, the insertion of the needle into the capsule wall is a manual mechanical process controlled by the patient. Alongside the puncture mechanism, the separation of the body from the capsule cap has also been proposed for opening the capsule (Twister^®^ Aptar, Crystal Lake, IL, USA, Rotahaler^®^ Cipla, Mumbai, India) as well as cutting the capsule with a blade (PowdAir^®^, H&T Presspart, Blackburn, UK). Figure 1 illustrates cDPIs with different capsule opening and piercing mechanisms.

Once the contents of the capsule have been made available for its release, the patient’s inhalation act generates turbulent air flows in the inhaler, which cause the capsule to move and release the powder contained therein. Around the 1980s, the Italian company ISF was the first to patent an inhalation device that caused the capsule to rotate around its minor axis [5]. To date, this spinning mechanism is still the most efficient in releasing and deaggregating the powder that leaves the capsule driven by centrifugal force [6]. This capsule movement was then included in improved Plastiape devices and in some developed later by other companies.

DPIs are mainly used in the treatment of respiratory diseases such as asthma, COPD and, more recently, cystic fibrosis. The active medicament as a dry powder is delivered using a device that enables its aerosolization in a suitable aerodynamic size for lung deposition (less than 5 microns) and an adequate delivery to the lung. Currently commercially available DPIs are passive devices since they rely on the patient’s inspiratory effort to provide the required energy to overcome the interparticle forces; few DPIs are active devices since they use other sources of energy. Because DPIs breath-actuated devices, the need to synchronize the actuation with inspiration is eliminated. However, DPIs show a device-specific airflow resistance, and this often demands a relatively high inspiratory effort, which might be a hurdle for patient with severe asthma or chronic obstructive pulmonary diseases, the elderly or the very young [7]. Therefore, the performance of each DPI can be affected by the patient’s inspiratory flow, and the turbulence produced inside the device, which uniquely depends on the technical characteristics of the device.

In summary, DPIs have several advantages over pMDIs: they simplify the inhalation technique and reduce the necessity for the patient’s cooperation.

Moreover, they enable the administration and deposition of high drug doses within the lungs, thereby limiting the incidence of both local and systemic side effects. In the last decade, new dry powder inhalation products have gained approval for different diseases, such as the TOBI^®^ Podhaler^®^ (Mylan, Canonsburg, PA, USA) and Colobreathe^®^ (Teva, Tel Aviv, Israel), that offer a new therapeutic option for patients suffering from chronic *Pseudomonas aeruginosa* infections associated with cystic fibrosis [4]. In relation to the administration of an inhalation product with systemic activity (Afrezza^®^, MannKind, Westlake Village, CA, USA), a human insulin inhalation powder employing a small and easy-to-use dry powder inhaler was approved in 2014 by the FDA for type 1 and type 2 diabetes mellitus patients. DPIs are gaining in market share and will become the dominant player in future years. This growth is due to new developments along with better device engineering and advances in powder formulations [7].

A final consideration must be made for this type of inhaler with respect to their sustainability, i.e., the impact of their production and use on greenhouse gas emissions. Recently, it has been calculated that DPIs have a carbon footprint 18 times lower than pMDIs due to the absence of a propellant [8]. In this regard, it would be interesting in the future to compare the difference in carbon footprint between capsule and multidose DPI inhalers.

## 2. Types of Dry Powder Inhalers

DPI devices vary widely in design and can be classified as single-unit and multi-unit types of devices. In a single-unit dose inhaler DPI, the powder is loaded in a mono-dose compartment as a cartridge, or more commonly as a hard capsule. This category can be classified into three groups by the method by which the capsule shell is opened to release the powder: puncturing with needles, cutting with blades or detaching the cap from the body of the capsule. The first two can be further sub-divided by the number and types of the pins and blades used. The majority of DPIs in use today use sets of either two or eight pins to penetrate the capsule shell wall. During the aerosolization, airflow streams enter via appropriate device inlets in the capsule chamber. In this way, under the inhalation airflow, the capsule starts moving and the powder is released through the pierced holes. The size 3 capsule is the size most used in the pharmaceutical industry for the development of capsule-based DPIs; however, hydroxypropyl methylcellulose (HPMC) capsules of larger volume were recently investigated for their ability to deliver a high powder dose (120 mg) of tobramycin during a succession of inhalations [9].

Critical elements in capsule-based DPI efficiency are the choice of an appropriate device, formulation expertise, technology for precision encapsulation and optimum packaging. The capsules are opened by puncturing with needles, detaching the cup from the body of the capsule or cutting by thin blades in the device to release the powder formulation upon inspiration. Capsules pierced by needles must be capable of being punctured efficiently, without cracking and losing fragments. Depending upon the design, capsules may be rotated laterally or axially in a symmetric airstream to facilitate the release of the drug to the patient. Specific attributes and characteristics are required for the successful administration to the lungs of inhalation therapies by capsule-based DPIs. Powder emission from cDPIs is affected by intrinsic resistance of the device, capsule opening and motion such as rotation, shaking and vibration. Hole size, and the capsule chamber volume may also influence the performance of the product. The inspiratory flow necessary to achieve a therapeutic effect is critical with DPIs; however, most patients with severe respiratory diseases have a weak inspiration flow. For this reason, the good practice of making two separate inhalations from the same dose is often recommended to ensure the inhalation of the whole of the dose [10]. Despite this inconvenience, capsule-based inhalers have a very positive response at low flow rates.

All currently available passive DPI systems are driven solely by patient’s inspiratory effort to disperse drug powders. Airflow through the device creates shear and turbulence; when the patient activates the DPI and inhales, air enters into the powder bed, which is fluidized and directed to the patient’s airways. Drug particles are then separated from the carrier particles: the former are carried deep into the lungs, while the latter impact in the oropharynx and are cleared [11]. Different DPIs have different intrinsic inhalation resistances that govern the resulting peak inhalation flow generated by the patient. This implies that a threshold inspiratory force is required to aerosolize, de-agglomerate and disperse the powder formulation and to achieve an effective drug deposition. The specific resistance to inhalation of each depends of the physical design of the device and is measured as the square root of the pressure drop across the device divided by the flow rate through the device. The current DPI designs have airflow resistance values ranging from about 0.02 to 0.07 $\sqrt{kPa}\cdot {\left(L/\min\right)}^{-1}$ [12]. To produce a fine powder aerosol with increased delivery to the lung, DPIs with low, medium or high intrinsic resistance require inspiratory flows of >90 L/min, 50–60 L/min and <50 L/min, respectively. Notably, due to the increased pressure drop across the device, high resistance DPIs tend to produce a greater lung deposition than those with low intrinsic resistance [12]. Namely, the increase in resistance means that low air flow rates are reached inside the inhaler, and this leads to particles which, given their relative low speed, are less subject to impact mechanisms in the upper airways.

The Breezhaler device is an example of a capsule-based DPI characterized by a low internal airflow resistance with a value of 0.02 $\sqrt{kPa}\cdot {\left(L/\min\right)}^{-1}$. Because of its low intrinsic resistance, it requires high inspiratory flow rates (100 L/min) to obtain a 4 kPa pressure drop. The flow rate values that are precisely established to standardize the in vitro characterizations of the devices are not always achieved in real life. However, an efficient device must be able to maintain the predetermined performance even at flow rate values around the optimal range. In this regard, Breezhaler delivered consistent doses even to COPD patients who generated a peak inspiratory airflow of approximately 90 L/min through the device [13,14]. In general, patients prefer DPIs with low resistance to those with high resistance [15]. In addition, Janssens et al. [16] have shown that, irrespective of the presence of airway obstruction, 30% and 12.5% of an elderly population were not able to reach the minimum peak inspiratory flow of 45 L/min when using the medium- to high-resistance Turbuhaler DPI and the low-resistance capsule-based DPI Aerolizer. Keeping this in mind, patients would benefit the choice of low resistance DPIs, which are relatively insensitive to variations in peak inspiratory flow at low flow levels about 40–50 L/min. The ERS/ISAM taskforce on inhalers [17] recommends patients “*to inhale forcefully from the beginning of inspiration, as deeply as possible, and to continue to inhale for as long as possible*”. Indeed, with a DPI, forceful inhalation disperses the micronized drug from the lactose-based carrier into a fine particle dose. The turbulence of the air flow generated in the device is directly related to the resistance of the inhaler and to the flow rate generated by the patient's inhalation act. Turbulence is the driving factor for the deaggregation of the powder and the generation of the fine particle dose.

The higher the airflow, the higher the powder dispersion generating a fine particulate, although high airflow may lead to a higher deposition of the powdered drug in the large airways and, as a result, to a lower dose in small airways [12]. On the other hand, low airflow increase deeper lung deposition of the powdered drug, although too low an airflow (such as that occurring in patients with severe airway obstruction) can limit deposition by affecting powder disaggregation and dispersion [12].

Multi-unit DPIs deliver single doses from pre-metered blisters, disks, or tubes. Multiple-dose DPIs have a reservoir that contains a bulk amount of powder formulation and a mechanism to meter and deliver a single dose with each actuation by the patient [18]. The dose is ready for its extraction by the activation of the device, which requires few maneuvers by the patient. It is agreed that the simpler the device, the lower the risk of error during use. Capsule-based DPIs require that single doses are individually loaded into the inhaler immediately before use, a maneuver potentially inconvenient for some patients and that does not allow direct counting of the remaining doses.

Then, the powder content of the capsule must be made available for release by a second patient maneuver: by pressing specific buttons on the device, the capsule shell is perforated or opened. Hence, the inhalation process must be continued or repeated until the capsule is emptied depending on the patient’s breathing profile. This maneuver may result in under-dosing and high dose variability. In addition, properly loading the cDPIs requires a sequence of steps that may not be easy for children or for elderly patients with reduced dexterity. In contrast, a study on usability of the cDPI Breezhaler has shown that patients found the device comfortable and easy to use and were confident of the medication being taken correctly when using the device [19]; however, the methodology to assess preference and satisfaction for an inhaler was assessed for a limited time interval by means of a non-validated methodology. Asthma and COPD patients [20,21] displayed no or fewer errors when using multiple-dose DPIs, such as the Ellipta, which require three steps to take the medication compared to the capsule-based DPI Handihaler or Breezhaler, which require eight steps to inhale the medication. These findings suggest that a DPI with a more intuitive design and requiring fewer steps to take the medication could be suitable for most patients.

Whatever the type, the DPI is more complex than the conventional dosage forms; it is a combined delivery system where its overall clinical performance is affected by its three main players: the patient, the formulation and the device. For this reason, in the pharma sector, the development of this type of product has moved away from the traditional Quality by Testing towards the Quality by Design approach, by which the only way to assure the quality of a product is by controlling its manufacturing process [22,23].

## 3. Formulation Aspects of DPI

The device and formulation must be compatible so that the formulation is easily delivered to the patient’s lungs. The formulation for DPIs usually consists of the API alone or the micronized API and an inert carrier such as lactose or mannitol. DPI formulation needs to be easily emitted from the device; it is important that it remains free flowing from the manufacture stage to the inhalation by the patient. For these natures of blends, the relationship between the formulation and the device is very important and the properties of the capsule are essential. DPI formulations tend to have a hygroscopic characteristic and the presence of moisture can potentially cause a change in powder flow properties. In this respect, a primary packaging capable of protecting the API, formulation and device from environmental humidity is required. Usually, inhalation capsules are packaged in blisters due to the high protection of this packaging. However, in some markets, inhalation capsules are packaged in 30 single-unit pill containers (for example Rotacaps (Cipla, Mumbai, India). On the other hand, when the powder tends to degrade or decompose easily, blisters that contain aluminum both in the plastic part of the cavities and in the lidding sheet are used. Insulin spray-dried powder showed to maintain good aerodynamic performance when filled in HPMC capsules and packaged Alu-Alu blister up to 6 months at room temperature conditions [24]. These findings open up the possibility to administer the daily therapy of diabetic patients without the need to refrigerate the product.

One advantage of the use of hard gelatin capsules is the higher potential for oxidation in HPMC capsules than in hard gelatin capsules. Hard gelatin capsules have demonstrated excellent protection against oxygen in comparison with HPMC material. Although powder formulations are less of a concern in this respect, the formulator can easily overcome the issue by choosing the right packaging to protect the powder from oxygen [25].

For both types of capsules that are chosen, it is always recommended that chemical compatibility between the API, excipients and the capsule be established as a first step to ensuring a promising formulation.

## 4. Capsule for Inhalation: Composition and Production Aspects

**Figure 2:**
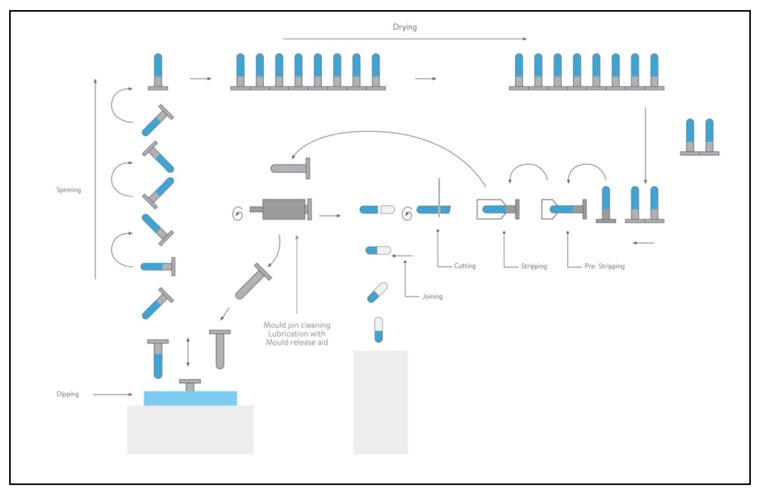
Capsule process production (courtesy supplied by Qualicaps).

There are two choices in capsule polymers that can be used for DPI formulations: hard gelatin capsules or HPMC capsules. Figure 2 shows the step of capsule process production, described here for the two types of material employed.

In the specific case of inhalation therapy, the capsule dissolution or disintegration test are not critical attributes. Further specifications such as moisture diffusion and permeability, physical and mechanical performance in a puncturing or cutting action or lubricant content on the inner surface should be evaluated. Finally, capsules for inhalation need a more stringent microbiological specification than that of standard oral capsules because their contents are directly inhaled into the lungs. The acceptance criterion based upon the total aerobic microbial count (TAMC) is <100 CFU/g according to Ph.Eur. 10th Edition.

Gelatin and HPMC are different with respect to their chemical and physical attributes, and the choice between the materials is ultimately based on the least amount of interaction between the formulation and capsule shell. Table 1 reports a list of commercial products where the powder dose is packaged with gelatin or HPMC capsules. Several sets of single or combined therapies for asthma or CPD are available, such as long-acting beta (2) agonist (LABA)/long-acting muscarinic antagonist (LAMA), LABA/inhaled chorticosteroids (ICS) or the recently approved Enerzair (Novartis) triple combination LABA/LAMA/ICS.

Gelatin is a biopolymer derived from the partial hydrolysis of collagen. Collagen is the predominant protein in mammalians and is the main component of animal bones and skins. Type I collagen, the main source of gelatin production, is composed of three so-called α chains, two identical α1 chains and one α2 chain that differs slightly in its amino acid composition. The three α chains are twisted around each other, forming the triple helical structure. The gelatin capsule shell shape is formed on lubricated stainless-steel mold pins mounted on steel bars at ambient temperatures that are dipped into a gelatin solution of a defined viscosity at 45–55 °C. The size and shape of the mold pins are specific for each capsule size and for the cap or body. Once the pins are dipped, a film forms on the surface, the pin bars are raised and they are rotated end over end to spread the film uniformly upon the pins as the bars are transferred to the upper deck of the machine. During this passage, cool air is blown over them to aid the setting of the film on the pins. Sets of pin bars are transferred by hydraulic pushers. The films are dried using large volumes of air at controlled temperature and humidity. When the dried capsule shells emerge from the last kiln, their moisture content is slightly above the upper limit. This allows the dried films to be removed from the pins without damage. The parts are then cut to the correct length and the caps and stripping bodies are joined together. In the gelatin capsules, the moisture content, acting as a plasticizer, is about 13–16%. Such a relatively high presence of water makes these capsules unsuitable for moisture-sensitive products where the humidity uptake changes the physico-chemical stability of the powder formulation.

Hard gelatin capsules have been successfully used in DPIs for more than 30 years, making them a standard choice for DPI development, given the wealth of data available on their use.

These were robust products and worked satisfactorily when their moisture content was within the specification of 13.0 to 16.0% [26]. However, they had the known drawback of becoming brittle following a loss of this moisture, which acts as a plasticizer of the shell. This phenomenon can occur in real life if the capsules are stored incorrectly and exposed to low-humidity conditions. In this case, the punctured holes by the inhaler are large and irregular; secondly, small pieces of shell wall may break off when they are cut, punctured or separated, and patients may inhale such small pieces. Typically, those fragments are too large to penetrate the lungs and impact mostly in the throat, which patients have reported as an annoyance. To prevent brittleness, the moisture contents of the drug and the capsule shell should be at equilibrium during filling. Moreover, modified capsules made of gelatin blended with a 5% polyethylene glycol 4000 as a plasticizer have been developed to further address this issue [27]. However, the problem of releasing shell fragments still exists. This is the case of Colobreathe^®^, a colistimethate sodium inhalation powder, indicated for the management of chronic pulmonary infections due to *Pseudomonas aeruginosa* in patients with cystic fibrosis. The powder dose of 125 mg is loaded in hard transparent PEG–gelatin capsules and aerosolized with the Turbospin inhaler using six or more inhalations [28]. Various instances have been reported of pieces of the capsule finding their way into a patient’s mouth and airways. Patients have complained of feeling these pieces on their tongue or in their airways.

In 2002, hard capsules made with HPMC were then proposed for use in DPI products to overcome the drawbacks related to gelatin; they have also demonstrated excellent characteristics in stability and aerosolization properties [29]. 

The new capsules retain their puncturing properties over a wider range of humidity than gelatin capsules because HPMC is more resistant to deformation. Compared to gelatin ones, HPMC capsules release a very small number of fragments from the shell walls when their moisture content falls because they do not become brittle [23,27].

HPMC capsules are highly chemically inert, which leads to far fewer incompatibilities, and contain much less water (about 4–6%) than gelatin ones, ensuring almost no brittleness upon storage at low relative humidity. Besides these technical issues, it should be considered that HPMC capsules are composed of vegetable sources. This material avoids both the risk of transmissible bovine spongiform encephalopathy and patient acceptance issues arising from vegetarian dietary restrictions and ethical matters.

Manufacturers of capsules have developed two main processes to produce HPMC capsules. The first is the “thermal gelling method” and involves dipping hot, metal mold pins, at ∼70 °C, into an HPMC solution at room temperature. The viscosity of HPMC solutions increases with a rise in temperature, forming a wet film on the pins, which are rapidly dried and set to create a stable film. The second method is “cold gelling” and involves adding excipients to the HPMC solution to produce a gelling system like that of gelatin, i.e., adding a gel network former, such as carrageenan or gellan gum, and a network promoter, such as potassium chloride or citric acid.

In this respect, capsules specific for inhalation use have been developed and proposed by different manufactures. Quali-V-I (Qualicaps, Tokyo, JP) is an HPMC-based capsule for inhalation, and its composition is different from that of other HPMC capsules on the market. HPMC is chosen using the right hydroxypropyl/methyl ratio with the correct molecular weight distribution. Carrageenan acts as a plasticizer and potassium chloride as the gelling promoter. This formulation enables capsules to be manufactured using the traditional cold gelled dipping process at ambient temperature.

Capsugel (Colmar, France), acquired recently by the Lonza group, developed a different HPMC cold-gelling system using gellan gum as the gelling agent and either ethylenediaminetetraacetic acid or sodium citrate as the gelling promoter, leading to Vcaps capsules. Apart from this production, Vcaps Plus DPI capsules are the only capsules available on the market with no gelling agents produced by the thermal-gelled technique.

Inhalation-grade HPMC capsules, due to the nature of raw material chosen for their puncturing properties [26], as a result have a slightly higher moisture content: 4.5–6.5% compared to 4.0–6.0% in oral pharmaceutical grade capsules [30]. In a recently published work, the aerodynamic performance of a formoterol fumarate powder blend was investigated by inserting the formulation into gelatin or HPMC capsules [31]. The cold-gelled HPMC capsules demonstrated a lower formoterol capsule retention and a higher delivered dose and Fine Particle Dose (FPD) than did the gelatin capsules. These differences in the aerodynamic performances were attributed to the difference in water content and to the increase in the hole diameter of the capsule that was cracked or fractured during the puncturing and inhalation step.

Similarly, it has been reported that the size and geometry of the hole significantly influenced the respirability of a ciprofloxacin powder stored in gelatin or HPMC capsules. When gelatin was used, although more formulations came out of larger irregularly shaped holes resulting in increased EF values compared to HPMC, the deaggregation of the particles was less efficient, which in turn reduced FPF values [32].

**Figure 3:**
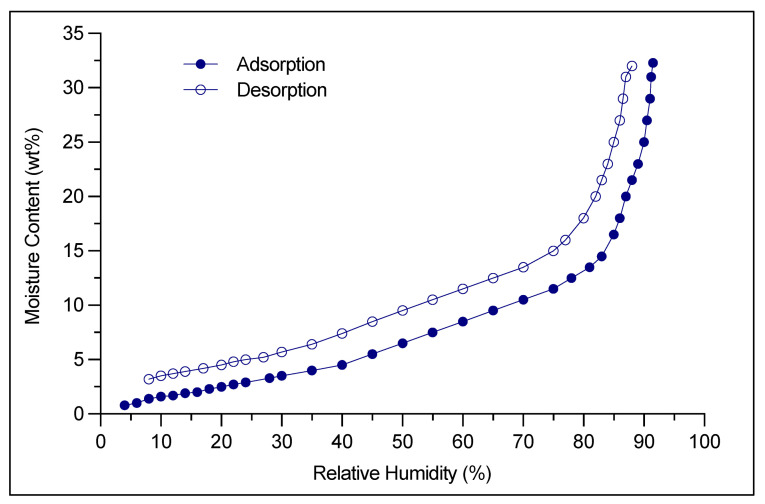
Water vapor adsorption and desorption isotherm curve obtained at 25 °C by Quali-V-I Extra Dry capsules (Qualicaps^®^, Tokyo, Japan). These data are obtained by means of a volumetric method as per USP41<1241> All data were obtained [33], https://ondrugdelivery.com/quali-v-extra-dry-a-novel-capsule-for-delivering-hygroscopic-pharmaceutical-drugs, accessed date: 5 October 2021.

Finally, for hygroscopic drug powders that are particularly sensitive to humidity, specific precautions and additional steps must be implemented post-capsule filling process. Formulation moisture content and filling room conditions are set precisely by the manufacturer and the final stage is drying the filled capsule. The drying step may take up to several hours (6–8 h, as well as 12–14 h) and represents a bottleneck in terms of timing within the drug manufacturing process [33]. Extra dry capsules instead of traditional HPMC capsules were proposed to improve product stability as well as leading to efficiencies and savings in drug product manufacture. In this regard, Quali-V^®^-I Extra Dry (Quali-V-I (Qualicaps, Tokyo, Japan) capsules have a very low moisture content between 2 and 3.5% and preserve this low range in ambient conditions ranging from 15 to 25% RH (Figure 3), which must be set up for low-moisture filling operations. This type of capsule, together with the specified environmental conditions, avoids the post-filling step of product drying, increasing the overall yield in cDPI production.

### 4.1. Effect of Moisture on Product Stability and Mechanical Performance

As discussed above, specific capsule attributes are required for the successful administration of inhalation therapies by capsule-based DPIs. These attributes include, for example, moisture diffusion and permeability from and through the capsule, and the physical and mechanical performance of the capsule in a puncturing or cutting action, including the hole shape, diameter and number of holes and lubricant content on the inner surface [23].

These capsule characteristics have recently been investigated by the scientific community and are reviewed here.

One of the most important differences between the two types of material of capsules is the amount of moisture. Moisture adsorption and desorption isotherms for empty HPMC and hard gelatin capsules have been investigated; for more moisture-sensitive formulations, HPMC capsules would appear to be a better choice than gelatin in terms of protection from moisture-induced deterioration [34]. In HPMC capsules, water does not act as a plasticizer and can be removed from capsule shells without a decrease in their physical properties. So, even when the moisture content was reduced to 1%, there was no increase in brittleness [31].

For highly moisture-sensitive formulations such as spray-dried lactose that was loaded into capsules, neither capsule types were capable of protecting the powder from induced solid state changes as a result of moisture uptake; thus, a protective secondary packaging is required for product stability during shelf life [31,34].

Moreover, studies with both of the polymer capsules containing the highly moisture-sensitive salicylic acid as a reference demonstrate the effect of HPMC capsules in reducing the water content of the drug if compared to the same formulation loaded into hard gelatin capsules [25]. When HPMC capsules were stored at RH not exceeding 65%, they had a low moisture content, of 3–8%, and these levels can be further reduced without any influence on their mechanical properties. Unlike gelatin capsules, these capsules did not become brittle in arid conditions.

**Figure 4:**
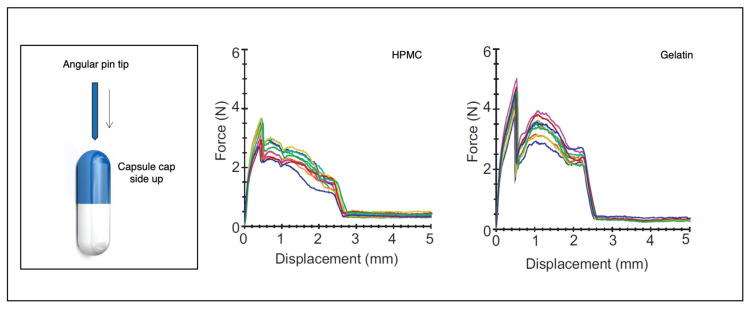
Single-pin puncture curves. Graphs of the displacement of a single angular pin versus the force for both hypromellose and gelatin capsules equilibrated at 33% relative humidity (Adapted from Ref. [36], published by Elsevier, 2013).

The physical and mechanical performances of the capsule in a puncturing or cutting action are other important attributes. Gelatin and HPMC capsules were punctured with insertion force measurement using a pin from an Aerolizer inhaler (diameter at approximately 0.06 mm). The result was that in HPMC capsules, the force after capsule puncture reduced by half and then increased to a second maximum as the pin shaft entered the hole. In gelatin capsules, the post-puncture force reduced to zero, indicating shell flaps losing contact with the pin (Figure 4). At lower moisture contents, both capsules were less flexible, although the HPMC capsules had similar patterns at both low and normal moisture contents, and gelatin capsules were less reproducible owing to complex interactions [31,35]. The force required for puncturing (max. force) was 2.82 ± 0.26 N for hypromellose Quali-V-I capsules and 4.54 ± 0.26 N for gelatin capsules stored at normal humidity [36].

### 4.2. Effect of Lubricant

HPMC capsules are made by a dipping process, and a surface lubricant for the mold pins is an essential processing aid for removing dried capsule shells. The lubricant is a mixture of food and pharmaceutical-grade materials registered with regulatory authorities, and the composition is proprietary for each capsule manufacturer.

The lubricant is applied to a circular foam roller that transfers the required amount to the pins as they pass underneath. The amount of lubricant is modulated by a pump, the flow rate from which can be adjusted using a pressure valve. An experimental work was performed to identify the machine settings to control the capsule for the inhalation manufacturing process and to produce capsules with the correct internal lubricant level [37]. In detail, an experiment was designed and performed to measure the effect of three critical machine factors: internal lubricant application pump-flow rate, pin position on a bar in the dipping pan and the time interval from the time of change of the application shells. It was demonstrated that the quantity and consistency of drug delivery from gelatin capsule DPIs were negatively affected by mold release lubricants used in capsule manufacturing. The lubricant was removed from assembled gelatin capsule shells using supercritical CO_2_ as a solvent, leaving dry and less retentive lubricant residue on the internal surface of the capsule. The treatment of capsules by supercritical CO_2_ brought about a large reduction in drug retention as well as a substantially higher and more reproducible fine particle mass [38]. The effect of the level of internal lubricant of HPMC capsules on the aerosolization properties of a salbutamol–lactose blend was also investigated [30]. In detail, the powder blend was loaded into HPMC capsules manufactured with three different lubricant levels and aerosolized via an eight-pin inhaler device. The study clearly indicates that the capsule lubricant level has an influence on deposition profiles and on the amount of drug remaining in the capsule and inhaler device after actuation: high lubricant levels are beneficial in decreasing drug deposition from capsules in the device. Furthermore, the medium and high level of capsule lubricant produced almost double the fine particle dose compared with the low level of lubricant. This effect was also related to the internal surface roughness of the capsule.

In addition to the importance of the internal lubricant in inhalation-grade capsules, the external lubricant content is a key factor in controlling and mitigating the electrostatic charge. Capsule-based DPI product performance can be influenced by electrostatic charging. Tribo-charging (increase in static charge) is a process of charge transfer during frictional contact and the subsequent separation of two solid surfaces, which can attract the API or formulation to the internal portion of the capsule, leading to a reduced dose delivered to the patient. It was recently demonstrated that gelatin capsules had a higher potential for tribo-charging than HPMC cold-gelled capsules. It was observed that all capsule materials tended to charge to a higher extent when in contact with PVC than with stainless steel. The different interactions between the capsule materials and water molecules entrapped in the shell were identified as being responsible for differing charging behaviors. Finally, it was shown that, depending on the capsule types, distinct environmental conditions were necessary to mitigate charging and assure optimal behavior of the capsules [39].

The addition and the type of external lubricant are beneficial not only in reducing the electrostatic charge of the capsule on filling, but also in increasing the in vitro respirability. In a recently published work, gelatin and HPMC capsules were used externally unlubricated and lubricated with carnauba wax, sodium lauryl sulphate or magnesium stearate [40]. This study investigated the potential connection between the mechanical properties of capsules coated with different external lubricants and the charging behavior of capsules during capsule filling and the delivered fine particle dose. Lubricated gelatin capsules showed a lower charge and delivered a slightly higher FPD compared to unlubricated capsules. In contrast, lubricated HPMC capsules showed a lower charge but delivered a significantly lower FPD compared to unlubricated capsules. Interestingly, both types of capsules lubricated with MgSt showed higher FPD as compared to other lubricants.

## 5. Conclusions

The therapeutic application of cDPIs began at the end of the 1960s, when the Spinhaler was introduced as the first DPI containing a powder formulation of broncho-active drugs in a gelatin capsule, which the patient loaded into the device prior to use. Since then, DPI systems have constantly evolved in technology and performance, a trend that continues. As for all DPIs, the most important advantage of capsule-based DPIs is that they are actuated and driven by a patient’s inspiratory flow, and, therefore, they do not require propellants to generate the aerosol, nor the coordination of inhaler actuation with inhalation.

Nowadays, several sets of single or combined therapies are available in the form of capsule-based DPIs, such as LABA/LAMA, LABA/ICS or LABA/LAMA/ICS. This type of product is widely used by patients worldwide and it is crucial that they are well trained in the use of them. These devices can lead to a high risk of making errors by the patient since they require some steps for their preparation and drug inhalation. On the other hand, because of their design, cDPIs entail a decreased risk of overdosing or double activation.

cDPIs have the capability of dispersing a high amount of drug by increasing the capsule volume, and the dose can be inhaled in consecutive inhalations. Therefore, the capsule would appear to represent a key element to metering and aerosolizing discrete quantities of the total dose to be administered. Such an approach would certainly appear to be more acceptable and more convenient to the patient, entailing several low-dose inhalations rather than a single one involving the inspiration of a large dosage.

Besides the inhaler features, this review has focused attention on the role and on the importance of the capsule. The ideal capsules for inhalation must be able to meet the following criteria: capsule shells must be capable of being either punctured or cut with the minimum of shell particles being shed; in the case of pin puncturing, the flaps produced must stay attached, remain open and not re-close or obstruct the powder emission; powders should empty from the shell with the minimum of retention and there should be a minimum of interaction between the shell and the fill material. These factors are influenced by the material they are made of as well as by the capsule moisture content and the level of internal/external lubricant. Finally, a reduction in shell moisture content should not cause capsule brittleness.

## Figures and Tables

**Table 1:** Currently marketed single-dose capsule-based DPIs. The list is organized in alphabetical order according to the marketed device employed for product aerosolization.

Device	Company	Commercial Product Name (Drug Delivered)	Type of Capsule
Aerolizer^®^	Novartis	Foradil (FF) Foradil Combi (FF, SS) Miflonide (BUD)	Gelatin
Breezhaler^®^	Novartis	Atectura (IDC, MF) Enerzair (IDC, GPB, MF) Miflonide (BUD) Onbrez (IDC) Seebri (GPB) Ultibro (IDC, GPB)	HPMC HPMC Gelatin Gelatin Gelatin HPMC
Handihaler^®^	Boehringer Ingelheim	Spiriva (TB)	Gelatin
Podhaler^®^	Mylan	TOBI (Tobramycin)	HPMC
Powdair^®^	H&T Presspart	Ventofor Combi Fix (BUD) (FF)	Gelatin
Rotahaler^®^	Cipla	Asthalin (SS) Budecort (BUD) Duolin (LS-IPB) Duova (TB, FF) Foracort (FF, BUD) Levolin (LS) Seroflo (SX FP) Triohale (ciclesonide, FF, TB)	- - Gelatin Gelatin Gelatin Gelatin Gelatin Gelatin
RS00	Kleva	Forcap (FF)	Gelatin
	Zentiva	Formolich (FF)	
	Italchimici	Kurovent (FF)	
RS01^®^	Adamem	Zafiron (FF) Fluxiton (FP)	Gelatin HPMC
	Allertec Hellas	Formaxa (FF)	Gelatin
	Baush Health	Forastmin (FF)	HPMC
	Chiesi Farmaceutici	Bronchitol (Mannitol)	Gelatin
	Deva	Brontio (TB) Foterol (FF) Foterol-B (FF, BDP) Respiro (FP, SX) Rolasym (FF, BUD) Sebraler (GPB)	Gelatin
	Exeltis	Fludalt Duo (FP, SX) Tioumit (TB)	HPMC Gelatin
	Galephar Nederland	Busalair (BUD, SX)	Gelatin
	Lek-Am	Pulmoterol (SX)	Gelatin
	Lupin	Budamate Forte (FF, BUD) Budate (BUD) Duomate (FF, BDP) Esiflo (FP, SX) Formoflo (FF, FP) Lupinhaler (TB) Salbair (LS) Salbair-I (LS, IB)	Gelatin
	Polpharma	Oxodil (FF)	HPMC
	Stada	Formoterol (FF)	HPMC
Spinhaler^®^	Aventis	Sodium cromoglycate	Gelatin
Turbospin^®^	Teva	Colobreathe (colistimethate sodium)	Gelatin
Twister^®^	Shanghai Sine Promod Pharmaceutical	Budesonide DPI	-
Zonda^®^	Teva	Braltus (TB)	HPMC

BDP: Beclomethasone dipropionate; BUD: Budesonide; FF: Formoterol fumarate; FP: Fluticasone propionate; GPB: Glycopirronium bromide; IDC: Indacaterol maleate; IP: Ipratropium bromide; LS: Levosalbutamol; MF: Mometasone furoate; SS: Salbutamol sulphate; SX: Salmeterol xinafoate, TB: Tiotropium bromide.

## Abbreviations

API	Active Pharmaceutical Ingredient
cDPI	Capsule-based Dry Powder Inhaler
COPD	Chronic Obstructive Pulmonary Disease
DPI	Dry Powder Inhaler
FPD	Fine Particle Dose
HPMC	hydroxypropyl methylcellulose
ICS	inhaled chorticosteroids
LABA	Long-acting beta(2) agonist
LAMA	Long-acting muscarinic antagonist
pMDI	Pressurised Metered Dose Inhaler
